# Complete Pectoralis Major Tendon Rupture in a Calisthenics Athlete: A Case Report

**DOI:** 10.5704/MOJ.2103.019

**Published:** 2021-03

**Authors:** CH Choo, WN Ng

**Affiliations:** 1 Department of Orthopaedic Surgery, University of Malaya, Kuala Lumpur, Malaysia; 2 Department of Orthopaedic Surgery, Sunway Medical Centre, Bandar Sunway, Malaysia

**Keywords:** pectoralis major rupture, callisthenics sports, rehabilitation, pectoralis major repair, y-knot anchor suture

## Abstract

We report a rare case of pectoralis major rupture during a body weight calisthenics exercise that was treated surgically. We highlighted the rehabilitation protocol which enabled him to regain full strength and return to his sport in three months.

## Introduction

Calisthenics is derived from the ancient Greek words kallos and sthenos, which meant “beauty” for the former word and “strength” for the latter word. In simple terms, using bodyweight as resistance in order to develop physique^1^. The common calisthenics moves are pull-up, muscle-ups, front lever, back lever, push-ups, pull-ups, handstand, dips, etc. All of which requires a lot of strength and flexibility^1^. Pectoralis major (PM) rupture is a rare entity in literature and most incidences had been reported frequently in weight-lifting and contact sports such as rugby^2^. The most common causal movement to this injury is overloaded eccentric contraction of PM with the shoulder in extension, abduction and external rotation^2,3^. However, there have not been any documented report on pectoralis major injury resulting from bodyweight exercises. Acute repair of PM rupture yielded better outcome as compared to non-operative treatment in active individuals who wish to return to their sports^3^.

We report a rare case of PM rupture during calisthenics exercise treated surgically. We highlighted the rehabilitation protocol which enabled him to return to his sport in three months.

## Case Report

A 32-year-old healthy callisthenic athlete was practicing a static move named “Hafesto” using a single right arm. This move involved shoulder extension to 70°, shoulder abduction of 90°, shoulder external rotation 30° and elbow flexion of 90°. After warming up, he attempted the highly intense static move when he felt a sudden sharp pain over the right upper chest region while performing the move and had to stop due to the pain. Next day, the anterior shoulder area was tender, swollen and ecchymosed. His shoulder adduction, flexion and internal rotation were weakened and painful.

**Fig. 1:**
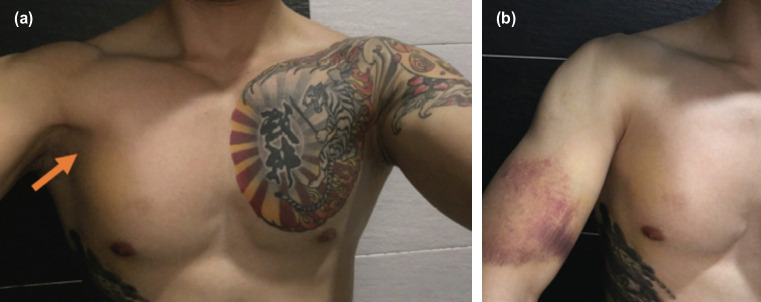
(a) The image of patient was taken 3 days after the PMT ruptured. Arrow showed a step over anterior axillary fold as compared to the left side. There was bruising and medialisation of PM muscle belly over right lower chest region. Also noted a “dropped nipple sign” as described by Funk. (b) There was large ecchymosis over right anterior arm. Most indicative of insertion tear of PMT.

**Fig. 2:**
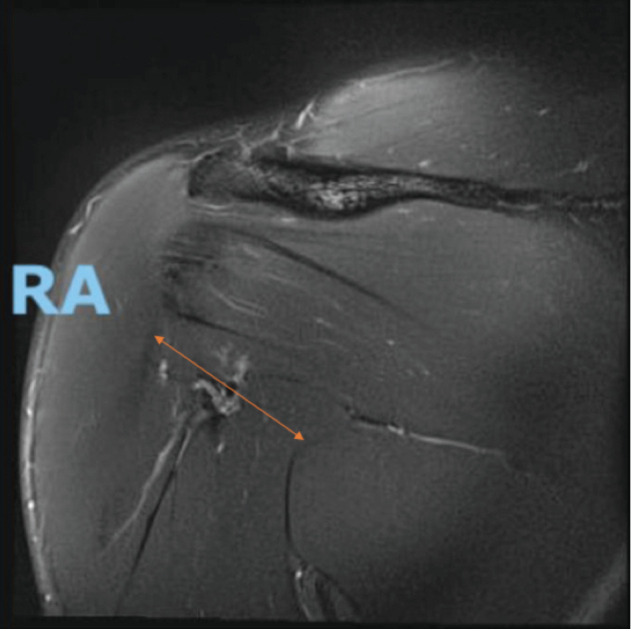
MRI coronal view of right chest showed complete PMT rupture and medialisation of muscle belly. Lower PM is retracted and leaving a 5cm gap.

On examination, the chest was asymmetrical, with notable defect over the right lower pectoral region and step over anterior axillary fold (Fig. 1a). There were also bruises over the right anterior deltoid which extended to the anterior biceps area (Fig. 1b). MRI right shoulder was performed which showed a complete pectoralis major rupture at the insertion. There was gap of 5cm pectoral muscles retraction and bunched up (Fig. 2). According to Tietjen classification^4^, he was classified as III D injury, when surgery is recommended. Surgical repair was conducted at two weeks after the initial injury as he wanted to return his sports without compromised strength.

The surgery was performed under general anaesthesia. The patient was positioned in the beach chair posture with a sandbag behind the scapula. Right shoulder and chest were prepped and draped with arm adducted and internally rotated under sterile conditions. A 5cm incision was made through a deltopectoral approach to expose the free ends of the pectoralis major tendon (PMT) and lateral lip of the bicipital groove. The pectoralis stump was identified and mobilised by releasing the surrounding adhesion. The PM stump was indicative of insertion rupture. The ruptured tendon was sutured using a modified Mason-Allen technique. The native insertion of PM tendon on humerus was debrided and burred.

Three unicortical drill holes were made along the insertion site. Free end of sutured PMT was pulled-through to the roughened surface of native insertion site and fixed with a Y-knot anchor suture [Conmed, New York, USA]. Wound washout was performed and closed with subcuticular sutures.

After surgery, accelerated rehabilitation was prescribed to assist recovery. Active range of movements returned to normal at eight weeks post-operation. Shoulder flexion 170°, extension 45°, abduction 180°, internal rotation up to T7 region and external rotation 45°. Visual analogue pain scale (VAS) returned to zero at week eight post-operatively. The Disabilities of the Arm, Shoulder and Hand (DASH) Score was zero at 10 weeks post-surgery for the patient. He was able to perform 50 push-ups in a minute by 12 weeks after surgery. He resumed his calisthenics exercises 12 weeks after surgery.

An isokinetic test was ordered to measure the recovery of strength and power objectively. The patient underwent isokinetic testing of his left shoulder at four months after surgery. At shoulder abduction of 180°, the abduction peak torque of affected shoulder showed 24.7% higher than left shoulder, at 40.1 NM, with a higher average power by 23.1 Watts. Whereas at 300° of shoulder abduction, the average power was 9.0 Watts more compared to the unaffected side Table I.

**Table I:** Isokinetic study of affected pectoralis major four months post-operation.

Movement		Abduction 180 Deg/Sec			Adduction 180 Deg/Sec			Abduction 300 Deg/Sec			Adduction 300 Deg/Sec		
Repetitions (180/180): 5		Uninvolved	Involved	Deficit	Uninvolved	Involved	Deficit	Uninvolved	Involved	Deficit	Uninvolved	Involved	Deficit
Repetitions (300/300): 15		Left	Right		Left	Right		Left	Right		Left	Right	
Peak Torque	N-M	32.2	40.1	-24.7	99.5	104.2	-4.8	24.5	23.6	3.9	89.5	86.9	3.0
Peak TQ/BW	%	53.7	66.9		166.0	173.9		40.9	39.3		149.4	145.0	
Max Repetition Total Work	J	35.5	43.3	-22.1	137.5	141.9	-3.2	21.2	17.6	16.8	104.5	96.3	7.8
Coefficient Of Variation	%	3.1	5.1		2.0	2.8		49.3	24.1		4.7	12.2	
Average Power	WATTS	45.5	56.0	-23.1	178.7	187.1	-4.7	18.2	19.9	-9.0	157.6	157.6	0.0
Total Work	J	165.1	197.6	-19.7	673.9	697.8	-3.5	171.3	177.0	-3.3	1418.3	1364.6	3.8
Acceleration Time	MSEC	150.0	120.0		100.0	90.0		240.0	210.0		150.0	160.0	
Deceleration Time	MSEC	80.0	90.0		150.0	130.0		110.0	100.0		160.0	150.0	
Range of Motion	DEG	101.8	101.8		101.8	101.8		101.0	100.9		101.0	100.9	
Average Peak Torque	N-M	30.3	37.2		97.5	101.7		17.4	18.4		84.8	82.0	
Agonist/Antagonist Ratio	%	32.3	38.5	G: 53.0				27.4	27.1	G: N/A			

Abbreviation; N-M: Newton-meter, TQ/BW: Torque/bodyweight, MSEC: milli-second, J: Joule, DEG: degree.

## Discussion

In view of patient’s fitness level, accelerated rehabilitation was planned in three phases: Reparative, Maturation and Resistance. Reparative phase (0-6 weeks) was designed to allow optimal tissue healing while retaining full range of movement in the shortest duration without compromising the surgical repair. The patient was kept on an arm sling with abduction pillow for three weeks to provide comfort and protection to the PMT. He was allowed to perform pendulum exercises twice a day and full range of movement exercises the for his wrist and elbow. At three weeks post-operation, once the fibrosis and scar formation began to mature at the surgical repair site, passive stretching of his shoulder was allowed. However, the movement was limited to flexion of 90°, abduction of 45°, and external rotation of 15°. Range of motion was gradually increased as tolerated by the patient. Full passive range of motion was achieved by end of six weeks.

During maturation phase (six weeks- full recovery), the aim of physical therapy was to recover functional movement of the shoulder without overloading or damaging the repaired PMT. This is done by improving mind-muscle connection, increasing endurance and correcting muscle imbalance (tonic and phasic). As the PM deconditioned after repair, patient’s ability to abduct or flex the shoulder were weaker than the contralateral side even though range of motion was the same. Active range of motion in all planes was encouraged during daily activity and progression weighted exercise regime was introduced. The program began with isometric exercises in multiplanar shoulder movements for the first two weeks (six – eight weeks after surgery). Patient performed isometric hold with shoulder in abduction, adduction, flexion, internal rotation and external rotation with a 5kg weight and gradually increased to 10kg by the end of two weeks. In the next two weeks, high repetitive of 2025 with lightweight dumbbells was performed at 60% to 70% of his one repetition maximum. The focus was to keep his muscles’ time-under-tension within three seconds; one second concentric action and two seconds eccentric action in each repetition. This optimises the vascularity and endurance of the muscles but minimises the stress on the healing PMT. From 10-12 weeks onwards, focus was directed at the tonic musculature to overcome muscle imbalance after disuse atrophy. Heavier load of 70% to 80% was applied over rotator cuff and scapula muscles resistance training.

Resistance phase was initiated at 12 weeks post-operation. The training was aimed at strengthening and remodelling the PMT. This was achieved by incorporating plyometric exercises that only involved bodyweight, i.e. explosive push up, pull up, and dips. Exercises were adjusted according to patient’s clinical symptoms and limitations as care was taken not to overburden the PMT. He was able to perform all the basic calisthenics exercises by the end of 14 weeks post-surgery.

He returned to calisthenics training with more advanced exercises by 16 weeks after surgery and resumed coaching at his fitness class. The key to his successful accelerated rehabilitation was his compliance to the rehabilitation program and applied scientific knowledge of tendon healing^5^. By closely monitoring his symptoms and response to physical therapy, we individualised the program to identify and resolve pain, joint stiffness and tissue guarding.

In conclusion, pectoralis major ruptures are rare with major contributions from sports-related injuries. This injury typically occurs in young active patients, especially heavy weightlifters. Although pectoralis major is not necessary for activity of daily living, an intact PM is important for patients who wish to continue weight lifting or return to sports. Therefore, surgical treatment is advisable for young athletic patients to achieve good results and return of function^3^. To the extent of our knowledge, this is the first report of a callisthenic athlete treated surgically for pectoralis major rupture and who returned to sports in a short amount of time through a structured rehabilitation program.
